# Cervical Sympathetic Blockade for Psychogenic Nonepileptic Seizures Associated With Post-traumatic Stress Disorder: A Case Report and Neurobiological Framework

**DOI:** 10.7759/cureus.116113

**Published:** 2026-09-11

**Authors:** Eugene Lipov, Siddharth Nair

**Affiliations:** 1 Pain Medicine, Stella Mental Health, Westmont, USA; 2 Anesthesiology, Midwestern University, Downers Grove, USA

**Keywords:** autonomic dysregulation syndrome, cervical sympathetic blockade, complex post-traumatic stress disorder, functional seizures, norepinephrine, psychogenic nonepileptic seizures (pnes), stellate ganglion block (sgb)

## Abstract

Psychogenic nonepileptic seizures (PNES), also termed functional seizures, are episodes that resemble epileptic seizures but occur without the abnormal ictal electrical activity characteristic of epilepsy. PNES is frequently associated with trauma exposure, post-traumatic stress disorder (PTSD), and abnormalities in autonomic regulation. Emerging evidence suggests that altered interactions among limbic, autonomic, and motor networks may contribute to symptom generation. Cervical sympathetic blockade (CSB), including stellate ganglion block techniques, has been investigated as an intervention for PTSD through modulation of sympathetic activity; however, CSB has not been established as a treatment for PNES.

This case report describes an unexpected and subsequently reproducible reduction in PNES following CSB performed for severe, treatment-refractory PTSD, and considers a possible relationship between sympathetic modulation and trauma-associated functional seizures.

A 27-year-old woman with severe PTSD and approximately 30 seizure-like or syncopal episodes per day was referred for CSB because of persistent PTSD symptoms despite prior conventional treatment. PNES was not the primary indication for the procedure. Ultrasound-guided CSB was performed at the C6 and C4 cervical sympathetic chain levels using 0.5% bupivacaine. Bilateral procedures were staged 24 hours apart as a safety precaution.

Following CSB, PTSD symptoms improved, while the reduction in PNES occurred on a more delayed time course. Seizure-like and syncopal episodes progressively decreased and ultimately ceased approximately two months after treatment. The patient subsequently regained functional independence. Approximately one year later, following recurrence of symptoms and repeat CSB, a similar delayed pattern of seizure reduction and elimination was observed again. No major procedural complications occurred.

The delayed but reproducible reduction and cessation of PNES following two separate courses of CSB suggests a possible association between cervical sympathetic modulation and functional seizure activity in a patient with severe trauma-related autonomic dysregulation. The mechanism is unknown, and the delayed response differs from the more rapid symptomatic improvement commonly reported following sympathetic blockade for PTSD. Potential downstream autonomic or neuroimmune effects may warrant investigation. This single-patient observation does not establish causality or support CSB as an established treatment for PNES, but it is hypothesis-generating and supports further prospective study of sympathetic modulation in carefully characterized patients with trauma-associated functional seizures.

## Introduction

Psychogenic nonepileptic seizures (PNES), also termed functional seizures, are paroxysmal episodes that resemble epileptic seizures but occur without ictal epileptiform activity on electroencephalography. PNES has an estimated prevalence of approximately 108.5 per 100,000 individuals [1], and long-term outcomes remain limited, with fewer than 40% of adults achieving sustained remission within 5 years [2]. The condition is associated with substantial psychiatric comorbidity, healthcare utilization, functional impairment, and reduced quality of life.

Increasing evidence supports a neurobiological model of PNES rather than one based solely on the traditional concept of conversion disorder. Neuroimaging studies demonstrate altered connectivity within corticolimbic, salience, and sensorimotor networks, including the amygdala, insula, anterior cingulate cortex, dorsolateral prefrontal cortex, basal ganglia, and sensorimotor regions [3]. Autonomic studies similarly demonstrate sympathetic predominance, reduced parasympathetic tone, peri-event tachycardia, and abnormal heartbeat-evoked potentials, reflecting altered cortical processing of visceral afferent input within the insular cortex [4]. Collectively, these findings support a model of abnormal integration among emotional, autonomic, and motor systems rather than volitional symptom generation.

Trauma exposure is common among individuals with PNES, with reported trauma histories ranging from 44% to 100% of cases; Fiszman and colleagues identified post-traumatic stress disorder (PTSD) as a significant risk factor in this population [5]. PTSD is itself characterized by amygdala hyperreactivity, impaired prefrontal inhibitory control, sustained noradrenergic activation, and chronic sympathetic overactivity [6]. Heightened central norepinephrine signaling reinforces hypervigilance, exaggerated startle responses, sleep disruption, and autonomic instability [7]--features that overlap mechanistically with PNES. This convergence suggests a shared limbic-autonomic substrate in which, in selected patients, trauma-related autonomic dysregulation may contribute to functional seizure expression.

Stellate ganglion block (SGB) and cervical sympathetic blockade (CSB), targeting the sympathetic chain at the C6 and C4 levels, are interventional techniques designed to modulate excessive sympathetic tone by anesthetizing the cervical sympathetic chain [8]. By attenuating central norepinephrine signaling and restoring autonomic balance, SGB/CSB may influence limbic hyperreactivity underlying trauma-associated conditions [7]. SGB has been investigated primarily for PTSD: in a randomized clinical trial, two right-sided SGB treatments administered two weeks apart produced significant reductions in Clinician-Administered PTSD Scale (CAPS-5) scores over eight weeks, although follow-up duration was limited [9]. In a comparative case series, patients receiving SGB combined with paroxetine demonstrated greater reductions in PTSD Checklist-Civilian Version (PCL-C) scores over six months compared with paroxetine alone, suggesting sustained benefit beyond pharmacotherapy [10]. CSB, which extends the blockade to the C4 level, represents a related approach intended to achieve broader cervical sympathetic modulation [8]. These procedures remain investigational with respect to PNES, and PNES itself was not the primary indication for treatment in the present case.

The patient described in this report was initially referred for treatment of severe, refractory PTSD. Psychogenic nonepileptic seizures occurred within the broader context of trauma-related autonomic dysregulation and were not the primary target of the procedure. The observed improvement in both PTSD symptoms and seizure-like episodes following cervical sympathetic blockade suggests that modulation of sympathetic-limbic circuitry may address overlapping pathophysiological mechanisms in trauma-associated PNES; this observation is hypothesis-generating and does not establish CSB as a treatment for PNES.

Patient selection for CSB

In our clinical practice, CSB is considered for carefully selected patients with clinically significant, persistent PTSD symptoms that have not responded adequately to conventional treatment, particularly when prominent hyperarousal and autonomic symptoms remain. Selection includes confirmation of the clinical indication, review of prior treatment response, assessment for procedural and local-anesthetic contraindications, and detailed informed consent regarding the investigational nature, potential benefits, and risks of sympathetic blockade.

## Case presentation

Methods

Cervical Sympathetic Blockade Procedure

Detailed informed consent was obtained before all cervical sympathetic block (CSB) procedures. The patient was placed in the supine position with the head rotated slightly to the contralateral side, and standard physiologic monitoring (heart rate, blood pressure, and oxygen saturation) was applied according to clinic protocol. The skin of the neck was prepared with chlorhexidine-isopropyl alcohol, and sterile ultrasound gel was applied. Using a broadband linear-array transducer (8-13 MHz), the neck was scanned in the transverse plane from the C6 to the C4 vertebral level.

The skin and subcutaneous tissue at the lateral neck injection site were anesthetized with 1.5 mL of 1% lidocaine. Using an in-plane technique under continuous real-time ultrasound guidance, a 22-gauge echogenic needle was advanced to a position immediately dorsal to the ventral fascia of the longus colli muscle and medial to the longus capitis muscle. After negative aspiration and with continuous patient monitoring, 0.5 mL of 0.5% bupivacaine was injected as a test dose. Following a 30-second observation period, an additional 4 mL was administered; after a further 30-second observation period and verbal confirmation from the patient that no concerning symptoms were present, a final 4 mL of 0.5% bupivacaine was injected slowly over approximately 1 minute, for a total injectate volume of 8 mL at the C6 level. The procedure was then repeated at the C4 level with an additional 4 mL of 0.5% bupivacaine.

The contralateral (left-sided) CSB was performed 24 hours later rather than during the same session, as a safety precaution. Staging the bilateral procedures was intended to reduce the risk of simultaneous bilateral recurrent laryngeal nerve involvement, which could result in bilateral vocal cord paralysis and clinically significant respiratory compromise. This same staged protocol, including technique, dosing, and 24-hour interval, was repeated for the second (bilateral) treatment course performed approximately one year later.

All procedures were performed at an outpatient interventional pain facility; the patient was not hospitalized for either treatment course. Clinical follow-up was performed at one week, one month, and two months after each procedure, with additional follow-up as clinically indicated thereafter.

Current Case

The patient was a 27-year-old woman with established diagnoses of post-traumatic stress disorder (PTSD) and psychogenic nonepileptic seizures (PNES), referred for further evaluation after failure of conventional medical and behavioral therapies. Prior to referral, she underwent comprehensive neurologic and autonomic evaluation. Electroencephalography demonstrated no epileptiform activity, and tilt-table testing was negative for postural orthostatic tachycardia syndrome (POTS). She completed a course of cognitive behavioral therapy with minimal clinical improvement. Pharmacologic management included clonazepam, prazosin, and a muscle relaxant (cyclobenzaprine), none of which produced sustained benefit. Her clinical course was complicated by multiple hospital admissions related to recurrent seizure-like and syncopal episodes.

At presentation, the patient reported approximately 30 seizure-like episodes and 8 syncopal events daily. Recurrent falls necessitated the use of a protective helmet. Her PTSD Checklist for DSM-5 (PCL-5) score, obtained one week prior to the first CSB, was 55 [11]. Because of the frequency and severity of events, she required continuous 24-hour supervision and was unable to function independently.

A right-sided CSB was performed at the C6 and C4 levels under ultrasound guidance, as detailed earlier. Following the procedure, she experienced a substantial reduction in seizure frequency and improved overall stability; within one month, she had become seizure-free, and her PCL-5 score had decreased to 31. Over the subsequent year, partial recurrence of both PTSD and seizure-like symptoms was noted. At the 1-year follow-up, her PCL-5 score had risen to 45, and bilateral CSB was performed using the same protocol described above.

Approximately 2 months after the bilateral procedure, the patient reported complete cessation of seizure-like and syncopal events, with her PCL-5 score again decreasing to 31. At the six-month follow-up, she no longer required supervision, had regained independence in activities of daily living, and had resumed driving. PTSD-related flashbacks and nightmares were markedly reduced. At the last follow-up, she remained on low-dose clonazepam and prazosin. No major procedural complications occurred.

## Discussion

This case supports a neurobiologically grounded interpretation of PNES and suggests that targeted sympathetic modulation may offer therapeutic benefit in carefully selected patients. Rather than representing purely psychogenic conversion phenomena, PNES appears increasingly consistent with maladaptive limbic-autonomic integration.

Neuroimaging studies demonstrate altered connectivity within amygdala-insula-prefrontal networks [3]. These regions are central to emotional salience processing, interoception, and top-down inhibitory control. Autonomic findings, including sympathetic predominance and abnormal heartbeat-evoked potentials, provide objective physiologic correlates of this dysregulation [4]. Importantly, these same neural and autonomic patterns characterize posttraumatic stress disorder (PTSD), a condition strongly associated with PNES [5]. The overlap suggests a shared pathophysiological substrate involving heightened sympathetic tone and dysregulated central autonomic network activity.

Anatomical evidence further supports this framework. Westerhaus and Loewy demonstrated multisynaptic projections from the stellate ganglion to supraspinal autonomic and limbic structures using pseudorabies viral tracing [12]. Labeling was identified in the hypothalamus, central and basolateral amygdala, insular cortex, and infralimbic cortex, confirming integration of peripheral sympathetic structures with affective regulatory circuits. These findings provide structural validation that modulation of the cervical sympathetic chain may influence central limbic networks implicated in both PTSD and PNES. The same cortical and subcortical regions involved in trauma-related hyperarousal appear to participate in seizure-like motor expression in PNES, supporting the hypothesis that reduction of sympathetic tone could benefit both conditions.

From a mechanistic perspective, PTSD has been theorized to involve a cascade initiated by trauma-induced upregulation of nerve growth factor (NGF), promoting sympathetic sprouting and amplification of central noradrenergic signaling (Figure 1) [7]. Sustained elevation of norepinephrine reinforces amygdala hyperreactivity, alters insular-mediated interoceptive processing, and perpetuates sympathetic dominance. Excessive noradrenergic tone may lower the threshold for autonomic instability and stress-triggered motor phenomena. Cervical sympathetic blockade (CSB) may interrupt this cycle by attenuating central norepinephrine signaling, dampening amygdala hyperactivation, and facilitating restoration of autonomic balance (Figure 2) [7].

**Figure 1:**
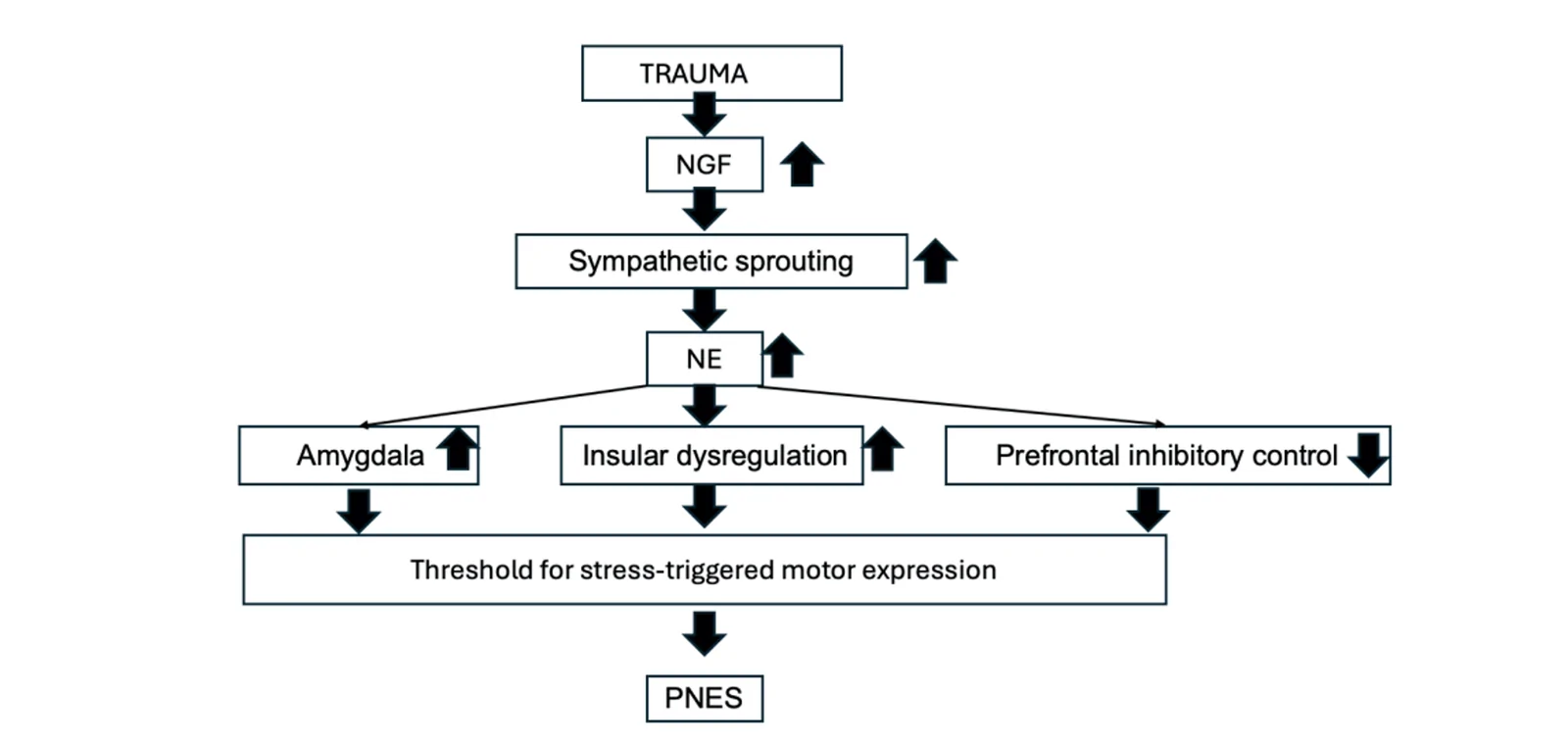
Limbic-autonomic mechanistic model of PNES Trauma exposure is hypothesized to upregulate nerve growth factor (NGF), promoting sympathetic sprouting and sustained elevation of norepinephrine (NE). Increased central noradrenergic tone enhances amygdala reactivity and contributes to insular dysregulation while reducing prefrontal inhibitory control. The combined effect lowers the threshold for stress-triggered motor expression, culminating in psychogenic nonepileptic seizures (PNES). Upward arrows denote relative increases (NGF, sympathetic sprouting, NE, amygdala activation, insular dysregulation), whereas the downward arrow at the prefrontal cortex denotes reduced top-down inhibitory regulation. The model illustrates maladaptive limbic–autonomic integration as a shared mechanistic substrate linking trauma-related sympathetic overactivation to seizure-like motor expression. Illustration created by the authors based on concepts described in [3,7,12].

**Figure 2:**
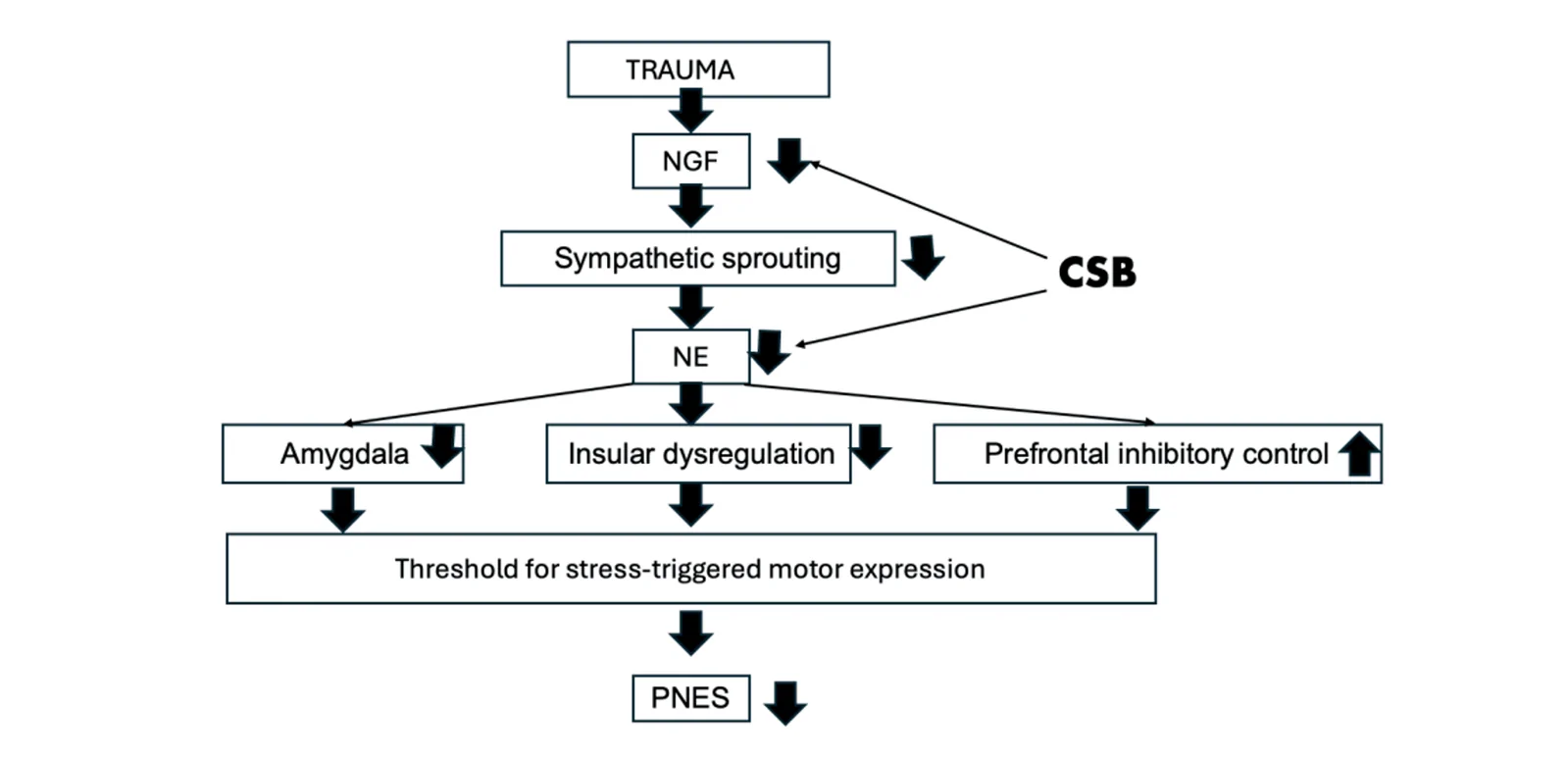
Proposed sympathetic-limbic-cortical model of stress-triggered motor expression in PNES and mechanistic target of CSB Psychological or physical trauma is hypothesized to increase nerve growth factor (NGF), promoting sympathetic sprouting and sustained elevations in norepinephrine (NE). Excess NE signaling alters limbic-cortical network dynamics, characterized by amygdala hyperreactivity and insular dysregulation, together with reduced prefrontal inhibitory control. This network imbalance lowers the threshold for stress-triggered motor expression, culminating in psychogenic nonepileptic seizures (PNES). Cervical sympathetic blockade (CSB) is proposed to interrupt this cascade by attenuating sympathetic outflow and reducing central NE tone, thereby restoring prefrontal inhibitory modulation and increasing the threshold for stress-induced motor manifestations. Illustration created by the authors based on concepts described in [3,4,6,7,12].

Notably, the clinical improvement observed in this case extended beyond the expected pharmacokinetic duration of local anesthetic. Such durability suggests that CSB may induce modulation of sympathetic plasticity rather than functioning solely as a transient conduction block. This interpretation aligns with theoretical models proposing recalibration of NGF-dependent sympathetic sprouting and central noradrenergic tone [7]. If trauma-related sympathetic overactivation contributes to both PTSD symptomatology and PNES expression, sympathetic blockade may act at a shared mechanistic node.

The delayed reduction in seizure activity observed approximately two months after CSB differs from the more rapid improvement frequently described following SGB for PTSD, and this discrepancy warrants explicit comment. In this patient, PTSD-related hyperarousal symptoms improved on a time course more consistent with the prior SGB literature, whereas the reduction and eventual cessation of seizure-like and syncopal events followed a distinctly delayed course that was not complete until approximately two months after each of the two treatment courses. The mechanism underlying this delayed response is unknown. However, a similar temporal pattern of seizure reduction and cessation was again observed following the second, bilateral treatment course approximately one year later, suggesting a reproducible association rather than a chance finding, although causality cannot be established from a single case.

One potential explanation involves sustained modulation of sympathetic-immune signaling rather than the immediate pharmacokinetic duration of the local anesthetic. Cervical sympathetic blockade has been reported to reduce circulating proinflammatory cytokines, including interleukin-6 (IL-6), and to modulate sympathetically driven neuroimmune signaling more broadly [7]. Elevated IL-6 has, in turn, been associated with the development of post-traumatic epilepsy following traumatic brain injury [13]. Delayed modulation of neuroinflammatory or neuroimmune signaling therefore represents one possible, though hypothetical, explanation for the observed time course; inflammatory biomarkers and catecholamine concentrations were not measured in this patient, so this mechanism remains speculative.

This case must be interpreted cautiously. Limitations include the single-case design, absence of quantitative autonomic and inflammatory biomarkers before and after intervention, and the potential contribution of placebo or expectancy effects, particularly given the absence of blinding or a control condition. The possibility of a placebo response cannot be excluded; however, in patients with trauma-related functional disorders, mind-body and expectancy-mediated mechanisms may themselves carry genuine therapeutic value rather than representing a purely inert confound, and this possibility does not necessarily diminish the clinical relevance of the improvement observed. Future investigations should incorporate standardized seizure frequency tracking, heart rate variability analysis, functional neuroimaging, validated PTSD symptom scales, and inflammatory and catecholamine biomarkers to help clarify mechanism and durability. Randomized controlled trials, ideally incorporating sham-block or delayed-treatment control arms, are necessary to determine efficacy, optimal laterality, and patient selection criteria.

Nevertheless, the observed parallel improvement in PTSD and PNES symptoms following cervical sympathetic modulation provides clinically relevant support for a shared limbic-autonomic model and warrants further systematic study.

## Conclusions

CSB may provide meaningful symptomatic and functional improvement in carefully selected PNES patients with trauma-associated autonomic dysregulation, although the delayed time course observed in this case and the single-patient design limit causal interpretation. In this case, right-sided CSB produced a substantial reduction in seizure frequency and improved PTSD symptom scores, and subsequent bilateral CSB was followed by complete cessation of seizure-like and syncopal episodes, with restoration of functional independence sustained through six-month follow-up after the second treatment course. Larger controlled studies are necessary to determine efficacy, durability, mechanism, and optimal patient selection criteria.
